# Integrated Lung, Diaphragm and Lower Limb Muscular Ultrasound: Clinical Correlations in Geriatric Patients with Acute Respiratory Illness

**DOI:** 10.3390/diagnostics15010087

**Published:** 2025-01-02

**Authors:** Nicoletta Cerundolo, Carmine Siniscalchi, Chukwuma Okoye, Simone Scarlata, Alberto Parise, Martina Rendo, Angela Guerra, Tiziana Meschi, Antonio Nouvenne, Andrea Ticinesi

**Affiliations:** 1 Department of Continuity of Care and Multicomplexity, Azienda Ospedaliero-Universitaria di Parma, 43126 Parma, Italy; ncerundolo@ao.pr.it (N.C.); aparise@ao.pr.it (A.P.); angela.guerra@unipr.it (A.G.); tiziana.meschi@unipr.it (T.M.); antonio.nouvenne@unipr.it (A.N.); 2 GRETA Research Group on Thoracic Ultrasound in the Elderly, Italian Society of Gerontology and Geriatrics (SIGG), 50129 Florence, Italy; chukwuma.okoye@unimib.it (C.O.); s.scarlata@unicampus.it (S.S.); 3 School of Medicine and Surgery, University of Milano-Bicocca, 20900 Monza, Italy; 4 Acute Geriatrics Unit, Fondazione IRCCS San Gerardo dei Tintori Hospital, 20900 Monza, Italy; 5 Unit of Internal Medicine, Fondazione Policlinico Universitario Campus Bio-Medico, 00128 Roma, Italy; 6 Research Unit of Internal Medicine, Università Campus Bio-Medico di Roma, 00128 Roma, Italy; 7 Primary Care Department, Parma District, Azienda Unità Sanitaria Locale di Parma, 43125 Parma, Italy; mrendo@ausl.pr.it; 8 Department of Medicine and Surgery, University of Parma, 43126 Parma, Italy

**Keywords:** muscle ultrasound, thoracic ultrasound, point-of-care, diaphragm dysfunction, sarcopenia, frailty

## Abstract

**Background/Objectives**: Point-of-care lung ultrasonography (LUS) represents an accurate diagnostic tool in older patients with respiratory failure. The integration of LUS with ultrasonographic assessment of diaphragm thickness and excursion, right vastus lateralis (RVL) muscle thickness and cross-sectional area (CSA) could provide real-time information on frailty and sarcopenia. The primary aim of this proof-of-concept prospective study was to evaluate clinical correlates of thoracic, diaphragmatic, and muscular ultrasound to characterize the associations between frailty, respiratory failure, and sarcopenia in older patients hospitalized for acute respiratory complaints. **Methods**: Each of 52 participants (age median 84, IQR 80–89 years old) underwent integrated LUS, diaphragm and RVL ultrasound examination upon admission (T0) and after 72 h of hospitalization (T1). LUS score was used to estimate lung interstitial syndrome severity. Diaphragm excursion, thickness, RVL thickness and CSA were measured following a standardized protocol. Frailty was assessed with the PC-FI (Primary Care-Frailty Index). **Results**: All patients exhibited multifactorial causes of respiratory symptoms. The LUS score on T0 predicted 3-month rehospitalization. Frail patients exhibited higher LUS scores on T1. Diaphragm excursion on T0 was reduced in patients with COPD and heart failure and in those developing delirium during hospitalization. Diaphragm excursion on T1 was negatively associated with PC-FI. Diaphragm thickness, RVL thickness, and CSA exhibited a positive association with obesity. Right vastus lateralis CSA on T1, however, was also negatively associated with PC-FI. **Conclusions**: Integrated lung, diaphragm, and RVL ultrasound shows clinical correlations with several aspects of frailty that may help to improve the management of geriatric patients with respiratory illness.

## 1. Introduction

Respiratory failure is the second leading cause of hospitalization among older patients. However, respiratory failure diagnosis may be challenging in this population due to patients’ unique characteristics [1]. In fact, older patients often exhibit atypical clinical presentations, abnormal responses to infection, distinct imaging findings, and frequent overlap of chronic conditions [1,2]. The challenges are especially pronounced in patients with higher degrees of frailty, sarcopenia, and cognitive decline [3,4].

Recent advancements in ultrasound techniques have proven increasingly valuable in this clinical context, demonstrating superior diagnostic accuracy compared to traditional radiological methods, particularly in the oldest old age group [5,6]. The integration of point-of-care ultrasonography (POCUS), performed at the bedside by expert clinicians, with each patient’s clinical presentation and history usually allows identification of the illness responsible for acute respiratory symptoms or, at least, the ruling out of the less probable diagnoses [7]. These advantages are emphasized in older patients with frailty and mobility limitations [6], where POCUS guarantees a higher diagnostic accuracy for the detection of bacterial pneumonia than traditional X-ray [8,9]. In older frail patients hospitalized for acute decompensated heart failure, POCUS has been also recognized as useful for tailoring diuretic treatment and optimizing discharge timing [10].

Recent data also suggest that common age-related respiratory conditions frequently causing urgent hospitalization, including chronic obstructive pulmonary disease (COPD) and congestive heart failure (CHA), are associated with diaphragm dysfunction, which is detectable with ultrasound [11,12]. Aging, in fact, may be associated with reduced diaphragm motility on both quiet and deep breathing, reduced thickness and thickening ratio [13,14]. Some authors have even proposed the concept of “respiratory sarcopenia” to define this condition, whose impact on the clinical course of acute respiratory illnesses remains unclear [15].

Despite the evidence suggesting that bedside lung and diaphragm ultrasound can provide prognostic information in selected clinical contexts, such as severe coronavirus Disease-19 (COVID-19) [16,17] and respiratory failure requiring mechanical ventilation [18], to date, no data exist on the association of lung and diaphragm ultrasound parameters with frailty, sarcopenia, and clinical outcomes in acute geriatric patients.

Apart from diaphragm involvement, sarcopenia is a very frequent condition in older patients who are hospitalized for acute respiratory illness [3,19,20]. Ultrasound of lower limb muscles has been proposed by the European Working Group on Sarcopenia in Older People (EWGSOP) as a valid screening tool for its assessment [21]. In different clinical settings, such as patients with critical illness, reduced lower limb muscle thickness and cross-sectional area (CSA) is associated with adverse clinical outcomes, including prolonged length of stay (LOS) and mortality [22,23].

Therefore, the integration of lung, diaphragm, and lower limb muscle ultrasound in older individuals hospitalized for acute respiratory illness has the potential of providing in vivo measurements of complex age-related phenomena, such as frailty and sarcopenia, that have a negative impact on clinical course and outcomes

The primary aim of this proof-of-concept study was to evaluate the utility of combining thoracic, diaphragmatic, and muscular ultrasound assessments to characterize the association between frailty, respiratory failure, and sarcopenia in older hospitalized patients.

Secondary aims included (i) assessing the predictive value of initial ultrasound measurements on 3-month rehospitalization and clinical deterioration, (ii) investigating associations between ultrasound-based assessments and frailty/sarcopenia metrics, (iii) exploring the influence of comorbidities including COPD and heart failure on diaphragmatic and RVL measurements, and (iv) observing changes in ultrasound parameters over 72 h to evaluate response to hospitalization.

## 2. Materials and Methods

### 2.1. Study Setting and Population

The study was conducted in an acute high-turnover ward of a Geriatric Department of a large teaching hospital in Northern Italy, serving a catchment area of >300,000 inhabitants. This ward mainly receives acute older patients from the ED, including a substantial number of subjects with respiratory illness.

Included in the study were patients aged ≥70 years old, admitted from the ED with suspected acute respiratory illness, irrespective of the presumptive diagnosis.

Exclusion criteria comprised suspected or confirmed SARS-CoV-2 infection, active respiratory cancer, terminal illness, previous thoracic surgery, previous intrapleural talc administration, known diaphragm paresis, right lower limb amputation, severe cognitive impairment, incapacity to maintain a sitting position, even with the aid of an assistant, or any other condition able to affect integrated ultrasonographic examination of chest, diaphragm and right lower limb.

All patients enrolled in the study signed an informed consent form, and the study was conducted in compliance with the Declaration of Helsinki and its later amendments. The study protocol was approved by the competent Ethics Committee (Comitato Etico Territoriale AVEN, Emilia-Romagna Region, Italy, ID 767/2022/OSS/AOUPR, date of approval 7 December 2022).

### 2.2. Study Procedures

Integrated ultrasound examination of lungs, diaphragm and right vastus lateralis muscle was performed by an expert operator, with at least five years of experience in point-of-care ultrasonography and who conducted more than 100 thoracic exams per year. All examinations were performed with an Esaote MyLab Alpha ultrasound (Esaote S.p.A., Rome, Italy), equipped with 3.5–5 MHz convex and 7.5–12 MHz linear probes, and with B-mode, M-mode and extended field-of-view (EFOV) functionalities. The first examination was performed for each participant within 24 h of ward admission (T0). A second examination was repeated, by the same operator and following the same methodology, after 72 h of hospitalization (T1), except in cases where patients were discharged earlier.

During ultrasound examinations, each hemithorax of participants was divided into six regions, considering the sixth rib and the parasternal, anterior axillary, posterior axillary and paravertebral lines as anatomical landmarks [24]. The regions were thus identified as anterior superior (between parasternal and anterior axillary lines, over the sixth rib), anterior inferior (between parasternal and anterior axillary lines, below the sixth rib), lateral superior (between anterior and posterior axillary lines, over the sixth rib), lateral inferior (between anterior and posterior axillary lines, below the sixth rib), posterior superior (between posterior axillary and paravertebral lines, over the sixth rib) and posterior inferior (between posterior axillary and paravertebral lines, below the sixth rib). LUS was performed systematically by scanning intercostal spaces of each of six regions of both hemithoraces, with both linear and convex probes. Patients were in the sitting position during examination, with the aid of a second operator in case of mobility limitations.

The resulting images were interpreted in compliance with international consensus guidelines, identifying signs of pleural effusion, pneumothorax, subpleural abnormalities, parenchymal consolidations, and interstitial syndrome [7,25]. Briefly, the presence of diffuse vertical artifacts (B-lines) was considered as indicative of diffuse interstitial syndrome, generally due to congestive heart failure or pulmonary edema. The presence of focal B-lines, subpleural consolidations with pleural line irregularities, or parenchymal consolidations with dynamic air bronchogram were considered indicative of bacterial pneumonia. Parenchymal consolidations with static air bronchogram were associated with atelectasis. Subpleural anechoic flaps were considered as indicative of pleural effusion, while absence of pleural line sliding or presence of lung point was associated with pneumothorax. The examination was considered normal in case of hyperechoic and regular pleural line showing sliding movements synchronous with respiration, absence of parenchymal consolidations, absence of pleural effusions, and presence of less than 2 B-lines per image. The final clinical diagnosis was formulated by integrating the results of ultrasound imaging with knowledge of the patient history, signs, symptoms and laboratory abnormalities. Normal ultrasound findings in patients with cough, dyspnea, or hypercapnic respiratory failure were considered indicative of COPD exacerbations. The severity of interstitial syndrome, if present, was graded with a method inspired by the one proposed by Soldati et al. for COVID-19 pneumonia [26,27]. More specifically, a score from 0 to 4 was assigned to each of the 12 thoracic regions in which ultrasound examination was performed. A score of 0 indicated absence of vertical artifacts or presence of isolated B-lines (≤2 non-confluent B-lines). Scores of 1 and 2 progressively indicated the presence of ≥2 vertical non-confluent B-lines for each ultrasonographic image, without or with irregularities of the pleural line, respectively. A score of 3 indicated confluent B-lines occupying the majority of the ultrasound image. A score of 4 was attributed to the white lung pattern.

Diaphragm examination was performed with patients standing in a semi-recumbent position, following a protocol formulated from literature analysis [28,29]. The linear ultrasound probe was put between the right anterior and mid-axillary line, in the eighth, ninth or tenth intercostal space, to identify the diaphragm zone of apposition. The diaphragm was visualized as the structure lying between two parallel hyperechoic lines, and its thickness was measured after activation of the M-mode ultrasound functionality, initially during quiet breathing and then asking patients to perform maximal voluntary inspirations. Diaphragm thickness on quiet breathing was measured three times at end-inspiration, corresponding to tidal volume (TV), and at end-expiration, corresponding to forced vital capacity (FVC). It was also measured three times on maximal voluntary inspiration, corresponding to total lung capacity (TLC). The average of the three measurements obtained for each parameter was used for statistical analyses. Then, the convex probe was put in the right subcostal position, in order to identify the liver parenchyma and the hyperechoic line surrounding it and corresponding to the right diaphragm hemicupola. Its excursions on quiet breathing and on maximal voluntary inspiration were then measured using the M-mode ultrasound functionality. Three measures of diaphragm excursion were acquired for each patient on both quiet breathing (TV) and maximal voluntary inspiration (TLC), and the average was used for the analyses.

Ultrasound examination of the right vastus lateralis muscle was performed as the completion of the chest ultrasound. The protocol by Ticinesi et al. was followed [30]. Briefly, the linear probe was put longitudinally on the center of the vastus lateralis belly, taking the transversal plane intersecting the distal third of the distance between great trochanter and lateral intercondylar space as the reference plane. Gentle tilting movements were performed by the operator to visualize the pennate architecture of muscle fascicles forming angles with the superficial and deep aponeuroses. The image was then frozen to measure vastus lateralis thickness as the distance between the two aponeuroses. This procedure was repeated three times, to acquire three thickness measures.

The EFOV function was then activated and the linear probe was rotated 90° counterclockwise, to acquire a transversal scan of the muscle on the reference plane. It was then moved transversally on this plane, from the lateral to the median end on the vastus lateralis muscle, in order to acquire a panoramic image of the whole cross-sectional area (CSA) thanks to EFOV. CSA was then measured on the ultrasound machine, using the freehand measure tool. The procedure was repeated three times also, to acquire three different CSA measures.

### 2.3. Data Collection

For each participant, data on the reason for hospital admission, chronic illnesses, weight, level of frailty, drugs being taken, vital signs and lab tests on hospital admission, including arterial blood gas analysis used for calculating the P/F ratio, were collected from medical records. Multimorbidity was measured by calculation of the Cumulative Illness Rating Scale Comorbidity Score (CIRS-CS) and Severity Index (CIRS-SI) [31]. Frailty was assessed using both the Clinical Frailty Scale (CFS) and the Primary Care-Frailty Index (PC-FI) [32].

The ultrasound parameters collected for each participant included the presence of specific abnormalities (i.e., pneumothorax, pleural effusion, bacterial pneumonia, other consolidations, interstitial syndrome), the extension of pleural effusions, measured by counting of the involved intercostal spaces, and the LUS score. Furthermore, the average measurement of diaphragm thickness on maximal voluntary inspiration (corresponding to TLC), end-inspiration on quiet breathing (corresponding to TV) and end-expiration on quiet breathing (corresponding to FRC), diaphragm excursion on quiet breathing and maximal voluntary inspiration, and vastus lateralis muscle thickness and CSA, were considered for each examination. Diaphragm thickening ratio was calculated as the difference between diaphragm thickness at end-inspiration and at end-expiration on quiet breathing, divided per thickness on end-expiration on quiet breathing. Variations between T1 and T0 were also considered for each parameter. LUS score on T0, LUS score on T1, diaphragm excursion on quiet breathing at T0 were dichotomized according to their medians in upper and lower values.

The study outcomes included respiratory failure needing NIV or high-flow nasal cannula (HFNC) during hospitalization, duration of oxygen treatment support or NIV, delirium during hospitalization, LOS, hospital mortality, and rehospitalization after 3-month follow-up for those discharged alive. The latter endpoint was assessed with a telephone interview with the patient or his/her caregivers. Delirium was diagnosed in compliance with the Confusion Assessment Method (CAM).

### 2.4. Statistical Analyses

Data were expressed as median and interquartile range (IQR) or percentage. Comparisons between demographic, clinical, anamnestic, ultrasound variables and endpoints were made after dichotomization of study population according to the medians of the ultrasound variables of interest. Comparisons between the characteristics of patients who experienced worsening of LUS score on T1 and those who experienced improvements, and between those in whom novel ultrasound abnormalities were detected on T1 with respect to T0 and those whose lung ultrasound abnormalities remained stable, were also performed. LUS scores, measures of diaphragm excursion, and their variations between T1 and T0 were also stratified by PC-FI categories. The Mann–Whitney test for continuous variables, Chi-square test or Fisher’s exact test for dichotomous variables were used for these analyses. The Jonckheere–Terpstra test was used to calculate the *p* for the trend of ultrasound variables across different PC-FI categories. Pearson correlation, linear or logistic regression were used to verify the clinical and anamnestic parameters associated with ultrasound variables.

The predictive capacity of ultrasound variables, in particular LUS score, for all the study outcomes was assessed with receiver operating characteristics (ROC) analysis and calculation of the area under the ROC curve (AUC). The Youden test was used to identify the optimal threshold value. ROC analysis was also used to identify the capacity of lower limb muscle ultrasound parameters to categorize participants as obese or non-obese. The cut-off of vastus lateralis CSA, identified with the Youden test, was also used to categorize participants as having a high or low CSA, and comparisons between these two groups were made using the statistical tests mentioned above.

Statistical analyses were performed with the SPSS software (v.29, IBM, Armonk, NY, USA). *p* values < 0.05 were considered as significant.

## 3. Results

### 3.1. General Characteristics of Participants

Fifty-two patients (25 F, 27 M; age median 84 years old, IQR 80–89) were enrolled in the study. Their baseline characteristics and outcomes are summarized in Table 1.

Overall, patients were multimorbid (CIRS-CS median 11, IQR 8–14) and frail (CFS median 4, IQR 3–6; PC-FI median 0.24, IQR 0.16–0.36). The causes of acute respiratory symptoms were multifactorial, with consistent overlaps among diagnoses (47% bacterial pneumonia, 49% non-inflammatory parenchymal consolidations including atelectasis, 67% pleural effusion, 90% interstitial syndrome). No participants had signs of pneumothorax at T0. Interstitial syndrome was detected, even in patients without a previous diagnosis of heart failure. Data on the results of ultrasound evaluation performed on T1 were available for 50 participants. Three patients died during hospitalization (6%), while seven patients were readmitted on 3-month follow-up. No patients were lost from the study or died during the follow-up period.

### 3.2. Lung Ultrasound

**Figure 1:**
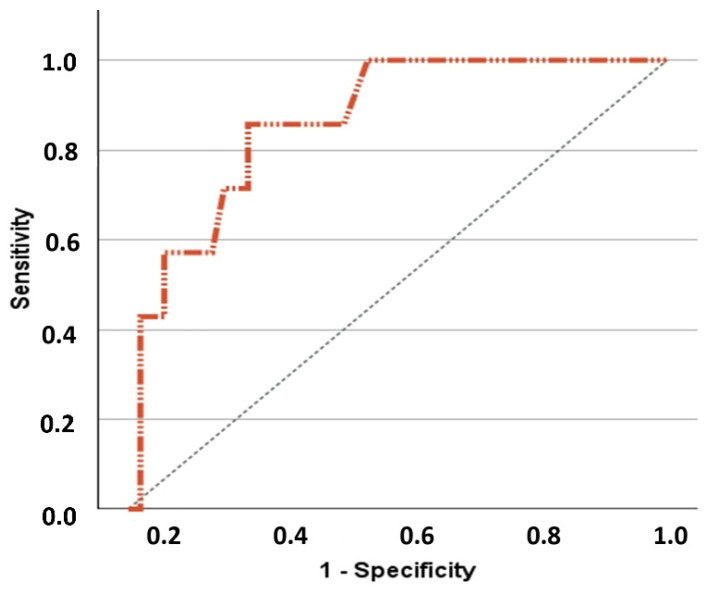
Receiver operating characteristic (ROC) curve showing the predictive capacity of lung ultrasound (LUS) score measured within 24 h of admission (T0) to predict rehospitalizations after 3-month follow-up in the studied population of older subjects acutely hospitalized for respiratory symptoms, irrespective of the main diagnosis.

To explore the relationship between baseline LUS score and clinical variables of interest in the studied population, we compared the characteristics and outcomes of participants stratified by the LUS score median at T0, i.e., 11. The results are depicted in Table 1. Although patients with LUS score at T0 ≥ 11 had similar CIRS-CS, CFS, PC-FI scores and hospitalization outcomes, they experienced a significantly higher readmission rate after 3 months in comparison with those with LUS score < 11 (26% vs. 0%) (Table 1). A comparison between the LUS characteristics of patients who experienced readmission vs. those who were not readmitted is provided in Supplementary Table S1. Notably, readmitted patients had a higher T0 LUS score (median 25, IQR 18–30, vs. 10, IQR 4–17, *p* < 0.001). We subsequently performed ROC analysis to verify whether baseline LUS score was associated with 3-month readmission in the studied population and, if this was the case, identify the optimal cut-off for prediction. The ROC analysis retrieved significant results for 3-month readmission (AUC 0.865, 95% CI 0.743–0.988, *p* < 0.001), with an optimal cut-off of 17.5 (sensitivity 86%, specificity 78%), determined with Youden test (Figure 1).

**Figure 2:**
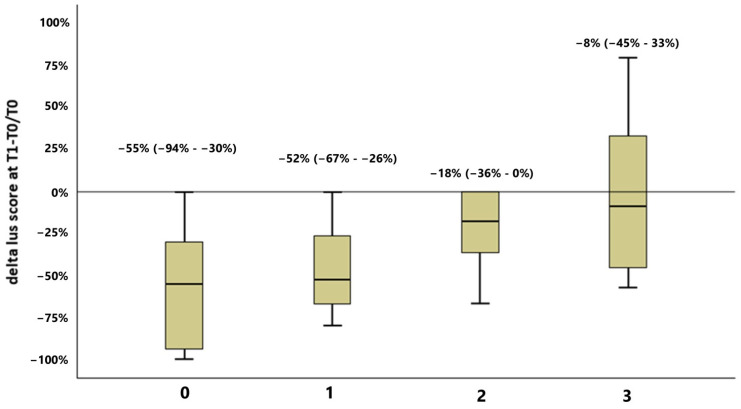
Comparison of LUS score variations between T1 and T0 (T1–T0/T0) after categorization of study participants into four groups of PC-FI (group 0: PC-FI < 0.20; group 1: PC-FI ≥ 0.20 and <0.25; group 2: PC-FI ≥ 0.25 and <0.40; group 3: PC-FI ≥ 0.40 (Kruskal–Wallis test *p* = 0.019; *p* for trend = 0.002).

LUS score improved at T1 in 33 patients (66%), while in the remaining 17 participants it was stable or worsened. The improvement was less pronounced for increasing categories of PC-FI, as shown in Figure 2 (*p* = 0.019 with Kruskal–Wallis test, *p* for trend = 0.001). A comparison of the characteristics of study participants stratified by LUS median on T1 is also provided in Supplementary Table S2. Patients with T1 LUS score ≥ 7 had a higher burden of frailty (PC-FI median 0.30, IQR 0.20–0.40, vs. 0.20, IQR 0.12–0.28, *p* = 0.013) than those with lower scores. Patients who did not experience improvements in LUS score at T1 had a higher prevalence of dementia (29% vs. 3%, *p* = 0.014), but did not experience readmissions (Supplementary Table S3). In 12 patients out of 50 (24%), a novel ultrasonographic diagnosis was made on T1, namely pneumonia or pleural effusion that were not visible on T0. This circumstance was associated with a higher CIRS-SI score, but not with baseline frailty, and implied a longer LOS, but not a higher rate of readmissions (Supplementary Table S4).

### 3.3. Diaphragm Ultrasound

The results of diaphragm excursion and thickness measurements in the whole population are depicted in Table 2. Patients with LUS score above the median had reduced diaphragm excursion at T0, but not at T1 (Table 2). Reduced diaphragm excursion on quiet breathing at T0 was associated with higher prevalence of COPD (58% vs. 24%, *p* = 0.014) and higher levels of frailty (PC-FI median 0.28, IQR 0.20–0.37, vs. 0.16, IQR 0.12–0.34, *p* = 0.034), and exhibited an inverse relationship with hemoglobin (Table 3).

The baseline clinical, anamnestic and laboratory factors independently associated with ultrasonographic measures of diaphragm excursion at different time points, determined with stepwise linear regression models, are depicted in Supplementary Table S5. Notably, diaphragm excursion on quiet breathing at T0 was inversely associated with hemoglobin levels (β ± SE −1.643 ± 0.432, standardized β −0.455, *p* < 0.001) and presence of cardiorespiratory comorbidity (β ± SE −5.547 ± 2.036, standardized β −0.325, *p* = 0.009), while diaphragm excursion on maximal voluntary inspiration was associated with procalcitonin levels at T0 (β ± SE 1.977 ± 0.769, standardized β 0.344, *p* = 0.014) and also with PC-FI at T1 (β ± SE −55.949 ± 23.122, standardized β −0.285, *p* = 0.023) (Supplementary Table S5).

**Figure 3:**
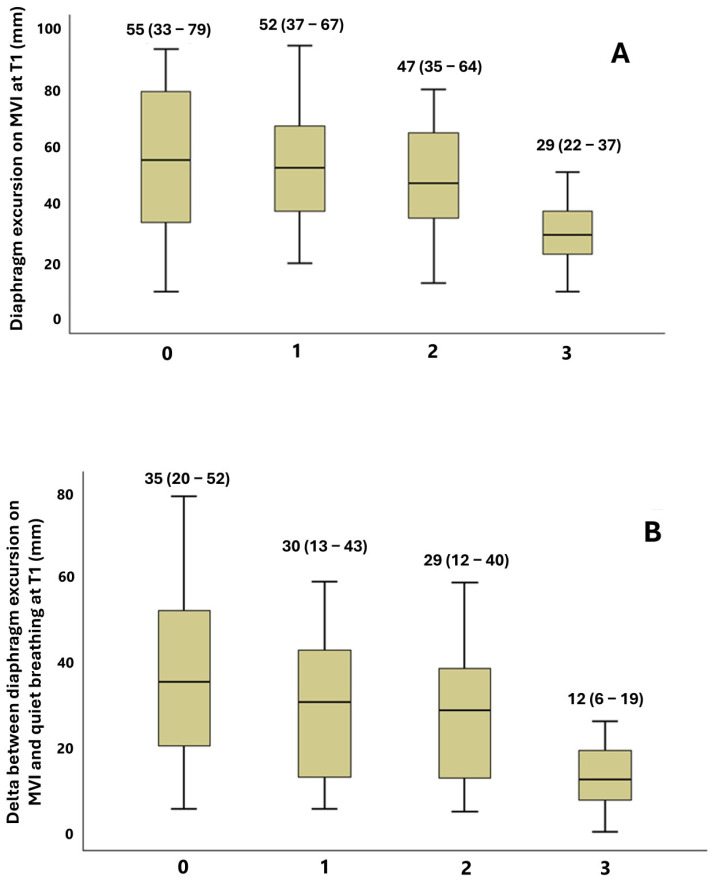
Comparison of diaphragm excursion at T1 (after 72 h of hospitalization), after categorization of study participants into four groups of PC-FI (group 0: PC-FI < 0.20; group 1: PC-FI ≥ 0.20 and <0.25; group 2: PC-FI ≥ 0.25 and <0.40; group 3: PC-FI ≥ 0.40). Panel (**A**) shows excursion on maximal voluntary inspiration (*p* for trend 0.019). Panel (**B**) shows the difference between excursion on maximal voluntary inspiration and excursion on quiet breathing (*p* for trend = 0.007).

Frailty was not related to diaphragm excursion at T0, whereas a decreasing trend of diaphragm excursion on maximal voluntary inspiration (*p* for trend = 0.019) and delta between maximal voluntary inspiration and quiet breathing (*p* for trend = 0.007) could be identified for increasing categories of PC-FI at T1 (Figure 3).

**Figure 4:**
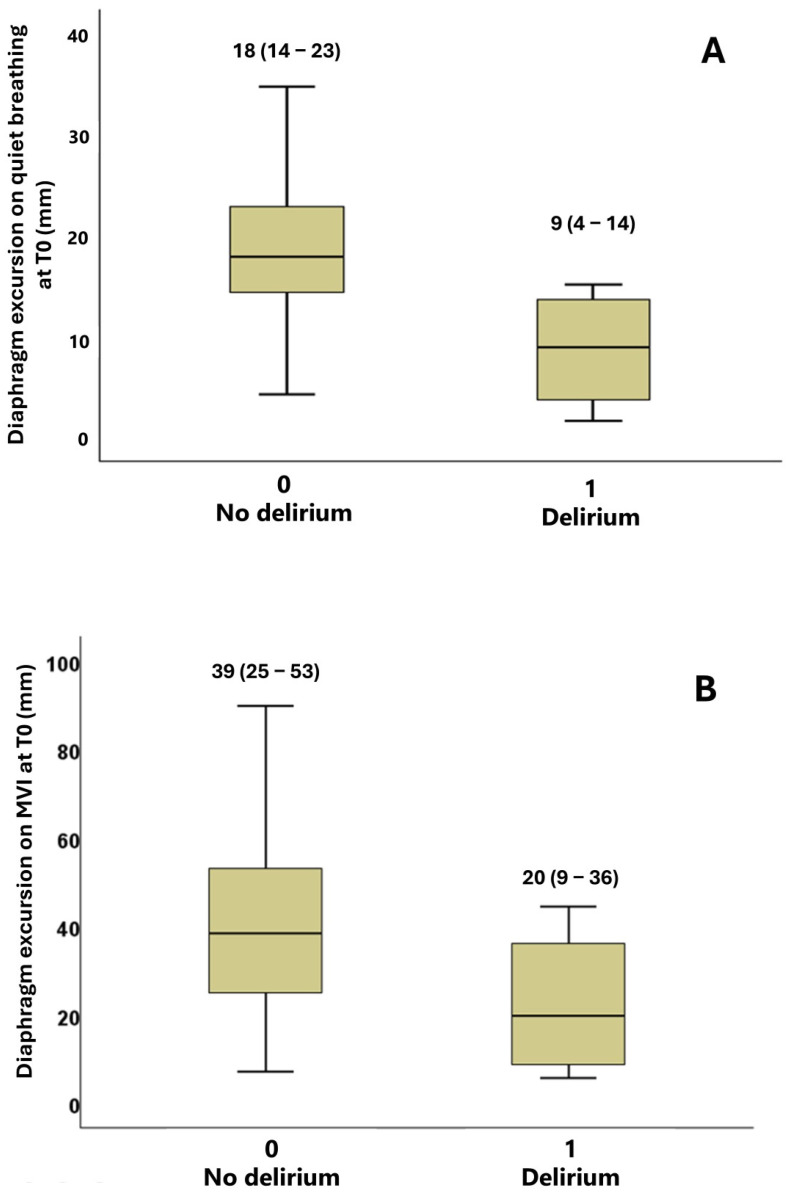
Comparison of baseline (T0) diaphragm excursion between patients who developed delirium during hospitalization and patients without this complication. Panel (**A**) shows excursion on quiet breathing and panel (**B**) shows excursion on maximal voluntary inspiration.

Diaphragm excursion was not associated with any of the considered clinical endpoints, except for delirium, which was more frequent in subjects with reduced excursion on quiet breathing at T0 (15% vs. 0%). Patients who experienced delirium during stay had reduced diaphragm excursion on quiet breathing at T0 (median 9 mm, IQR 4–14, vs. 18 mm, IQR 14–23, *p* = 0.009) and a tendency towards reduced excursion on maximal voluntary inspiration (median 20 mm, IQR 9–36, vs. 39 mm, IQR 25–53, *p* = 0.090) (Figure 4). In a logistic regression model, diaphragm excursion on quiet breathing at T0 was inversely associated with delirium (OR 0.767, 95% CI 0.609–0.965, *p* = 0.023).

Diaphragm thickness on different pulmonary volumes was unrelated to lung ultrasound findings (Table 2). At T0, diaphragm thickness was negatively correlated with age (thickness on TV: r = −0.460, *p* = 0.002; thickness on FRC: r = −0.336, *p* = 0.045) and positively correlated with the presence of obesity (thickness on TV: r = 0.376, *p* = 0.012; thickness on FRC: r = 0.333, *p* = 0.047; thickness on TLC: r = 0.329, *p* = 0.029). Diaphragm thickness at T0 on any pulmonary volume was able to discriminate between obese and non-obese participants on ROC analysis (Supplementary Table S6).

In obese participants, diaphragm thickness at T0, measured on both quiet breathing and maximal voluntary inspiration, also exhibited a significant inverse correlation with diaphragm excursion on quiet breathing (thickness on TV: r = −0.617, *p* = 0.032; thickness on FRC: r = −0.667, *p* = 0.018; thickness on TLC: r = −0.675, *p* = 0.011). Conversely, these correlations were not seen in non-obese participants.

Diaphragm thickness at any pulmonary volume, thickening ratio, and their variations were not associated with any of the clinical outcomes considered in the study.

### 3.4. Vastus Lateralis Muscle Ultrasound

Right vastus lateralis muscle thickness and CSA at both T0 and T1 were unrelated to lung ultrasound findings (Table 2). However, thickness at T1, but not at T0, exhibited a significant positive correlation with body weight (r = 0.495, *p* = 0.002) and obesity (r = 0.371, *p* = 0.008). Similarly, vastus lateralis CSA at T1 was positively correlated with body weight (r = 0.720, *p* < 0.001) and obesity (r = 0.475, *p* = 0.001). The presence of vastus lateralis CSA ≥ 9.91 cm^2^ at T1 was significantly able to discriminate between obesity and non-obesity in the studied population (Supplementary Table S6).

A comparison of the clinical and ultrasound characteristics of patients categorized according to this cut-off of vastus lateralis CSA at T1 is depicted in Supplementary Table S7. Interestingly, subjects with CSA < 9.91 cm^2^ at T1 had a higher burden of frailty (PC-FI median 0.28, IQR 0.19–0.40, vs. 0.20, IQR 0.12–0.24, *p* = 0.010), worse baseline respiratory conditions (P/F median 223 mmHg, IQR 184–306, vs. 324 mmHg, IQR 226–344, *p* = 0.031), and longer duration of oxygen support (median 7 days, IQR 4–11, vs. 4 days, IQR 0–7, *p* = 0.008).

## 4. Discussion

This study shows that the application of an integrated ultrasonographic examination of lungs, diaphragm and right lower limb muscles in group of older patients urgently hospitalized for unselected acute respiratory complaints is able to provide various clinical information improving the understanding of the interactions between acute illness and frailty.

First, LUS findings revealed that the cause of acute respiratory complaints in older patients is frequently multifactorial, with an important contribution of congestive heart failure, detectable on ultrasound as interstitial syndrome. This condition, whose severity in the acute phase can be graded with the LUS score, can either represent the main cause of dyspnea or a secondary condition complicating pneumonia, bronchitis, or COPD exacerbation. These concepts are coherent with the findings of the Atherosclerosis Risk in Communities Study and the Cardiovascular Health Study, where a high prevalence of multifactorial causes was detected in older participants with undifferentiated dyspnea [33,34]. LUS can thus represent a smart and reliable tool to understand the pathophysiology of acute dyspnea in geriatric patients and help clinicians to personalize treatments [6].

In this context, the LUS score is a quick and reproducible measure, providing not only diagnostic information on the presence of congestive heart failure, but also an estimation of severity of the illness and the risk of hospital readmission. The prognostic value of the LUS score has been demonstrated, to date, only in patients with severe forms of COVID-19 [16,17], and in patients with critical illness admitted to an ICU [35], but not in geriatric patients upon admission. Two studies, however, found that the systematic count of LUS B-lines, a parameter similar to the LUS score, on hospital discharge of older patients with decompensated heart failure was able to predict early readmissions [36,37]. Our data also suggest that in geriatric patients the association between LUS score and readmissions is maintained independently of the fact that heart failure represents the main cause of hospitalization.

The presence of COPD or heart failure was also associated with reduced diaphragm excursion in the studied population. In COPD, a reduction in diaphragmatic excursion can represent the effect of lung hyperinflation, and also exhibited a correlation with pulmonary volumes determined by spirometry in previous studies [38,39]. Furthermore, a recent study by Scarlata et al. showed that, in patients with congestive heart failure, there is a trend towards reduced diaphragm excursion with increasing NYHA class [12]. These phenomena may be amplified in older patients, who exhibit substantial overlap between the two chronic conditions.

Diaphragm excursion on quiet breathing was also inversely associated with hemoglobin levels in our study. The presence of anemia, a very frequent condition of multifactorial origin in geriatric patients, should be considered when interpreting diaphragm ultrasound findings, because it could mask the presence of a diaphragmatic dysfunction. To date, a relationship between diaphragm excursion, assessed by ultrasonography, and hemoglobin levels has been demonstrated only in adult patients with sickle cell anemia [40] and patients under maintenance hemodialysis [41]. Therefore, further studies should investigate the importance of this association, especially in older individuals.

Interestingly, our results suggest that reduced diaphragmatic excursion on admission was associated with incident delirium during hospitalization, albeit the sample size was not sufficiently large to draw solid conclusions about this relationship. Respiratory insufficiency, of which reduced diaphragm excursion represents a marker, is a well-known trigger of delirium, especially in patients admitted to an ICU and in older individuals with pneumonia [42,43]. In geriatric patients, delirium can also represent the main clinical presentation of acute respiratory conditions, as recent experience with the COVID-19 pandemic has demonstrated [44]. Although this association could be spurious, it remains extremely important in clinical terms, because the identification of ultrasonographic signs of diaphragm dysfunction may help to identify patients with a higher risk of delirium during hospitalization.

Conversely, ultrasonographic assessment of diaphragm thickness provided limited clinical information in the studied population, as it was influenced by the condition of obesity. Interestingly, obese subjects with increased diaphragm thickness on ultrasound also exhibited reduced motility. In obese individuals, the curvature of the diaphragm is modified in comparison with subjects with normal weight, causing a mechanical disadvantage that may be compensated by local hypertrophy [45,46]. Thus, the novel concept of “respiratory sarcopenia”, implying diaphragm dysfunction due to generalized loss of muscle mass and function [15], should take into consideration the particular characteristics of obese subjects, and ultrasound evaluation of diaphragm muscle thickness may not be reliable in subjects with sarcopenic obesity.

Obesity also affected vastus lateralis muscle ultrasound measures of thickness and CSA in the studied population. This circumstance may affect the capacity of lower limb muscle ultrasound to identify sarcopenia and sarcopenic obesity [47]. However, according to a recent study conducted in older patients with obesity, lower limb ultrasound was more accurate than bioimpedance analysis in detecting sarcopenia [48].

Lower vastus lateralis CSA was, in any case, associated with worse respiratory presentation in the studied population, identifying a subgroup of participants with a higher burden of sarcopenia, and therefore at risk for a more complicated clinical course [49]. Vastus lateralis CSA was also inversely associated with the level of frailty measured with PC-FI, reinforcing the concept that reduced muscle mass represents the biological substrate of frailty. In this regard, ultrasound implementation in clinical practice could represent a quick strategy to screen for sarcopenia and guide the identification of patients with frailty, as suggested by an increasing number of recent studies [50,51,52,53]. Furthermore, data from the literature suggest that lower limb muscle ultrasound can be also used to monitor muscle mass loss, and thus acute sarcopenia onset, in older patients with prolonged hospitalization [54]. In our study, no significant variations of muscle area were detected between T1 and T0, probably as a consequence of the short interval between the two assessments.

Our study also shows that frailty does not only influence lower limb muscle ultrasound findings, but has also significant consequences on parameters of lung and diaphragm ultrasound after 72 h of hospitalization. In fact, the LUS score was higher and diaphragm excursion on maximal voluntary inspiration was lower in subjects with frailty than in non-frail individuals. In older individuals, frailty interferes with the clinical course and prognosis of several acute and chronic illnesses [55,56], reducing the capacity of resilience of different body systems [57]. This effect has also been demonstrated in acute respiratory diseases, where the presence of frailty can slow down recovery time [58] and be associated with persistent inflammation, leading to further physical decline [59]. Therefore, the finding that frailer patients had more severe interstitial syndrome on T1 suggests that the response to treatment was impaired and recovery time from congestive heart failure features was prolonged by frailty. Similarly, the finding that frailer patients had reduced diaphragm excursion on maximal voluntary inspiration at T1 suggests that their recovery was slower also in terms of diaphragmatic function. Interestingly, in a recent study conducted in 64 older patients in an outpatient setting, reduced diaphragm excursion was associated with the presence of physical frailty and sarcopenia [60].

The finding that both LUS score and diaphragm excursion were less related to frailty at the moment of hospitalization (T0) suggests that, at this time, the effect of frailty on ultrasound parameters could have been masked by the effects of the acute illness. Therefore, ultrasound correlates of frailty and sarcopenia may be better assessed once the patient has passed the acute phase.

Several limitations should be considered when interpreting our findings. The most obvious ones are the small sample size, the absence of a formal assessment of sarcopenia or body composition, and the lack of assessment of respiratory muscle strength. Furthermore, leg dominance was not considered in muscle ultrasonography, mainly for technical reasons. In fact, the assessment of the left vastus lateralis muscle requires movement of ultrasound equipment and operator to the patient’s left side, which is impractical in hospitalized individuals, considering that diaphragm ultrasound is performed on the right side. However, recent research suggests that leg dominance may significantly affect the parameters measured during muscle ultrasound examination [61]. Lastly, combination of lung, diaphragm and muscle ultrasound parameters in a unique index was not possible, mainly because of the small sample size.

Despite these limitations, to our knowledge, this is the first study investigating the clinical correlates of an integrated lung–diaphragm–muscle ultrasound examination in older subjects with acute respiratory illness. The assessment of ultrasound parameters at two time points during hospitalization allowed us to identify their dynamic variations and to separate the effects of acute illnesses from the effects of pre-existing frailty. Therefore, the findings of the present study should be regarded as hypotheses generators for the design of future clinical studies in the field of geriatric ultrasound.

## 5. Conclusions

In a group of older patients acutely hospitalized for respiratory illness, the application of integrated bedside lung–diaphragm–muscle ultrasound provided a large amount of clinical information. In particular, the LUS score was associated with 3-month rehospitalizations and was significantly modified during the clinical course by the presence of frailty. Diaphragm excursion was reduced in patients with pre-existing COPD, heart failure, and frailty, and this dysfunction was associated with increased risk of delirium during hospitalization. Diaphragm thickness, however, was strongly influenced by obesity in the studied population. Reduced right vastus lateralis muscle CSA, corresponding to an increased odds of sarcopenia, was associated with more severe respiratory failure on admission, and with frailty.

## Figures and Tables

**Table 1:** General characteristics of participants and comparison after stratification for LUS score median at T0.

Parameter	Overall Population (*N* = 52)	Patients with LUS Score < 11 at T0 (*N* = 25)	Patients with LUS Score ≥ 11 at T0 (*N* = 27)	*p*
General and anamnestic characteristics				
Age, years	84 (80–89)	83 (79–86)	86 (81–89)	0.123
Females, %	48	52	44	0.594
Weight, kg	71 (63–81)	74 (62–90)	71 (62–79)	0.470
Chronic illnesses, number	5 (4–6)	4 (4–5)	5 (4–6)	0.313
COPD, %	40	40	41	0.958
Heart disease, %	73	68	78	0.437
Obesity, %	31	32	30	0.857
CKD, %	29	24	33	0.468
Dementia, %	12	16	7	0.411
CIRS-CS	11 (8–14)	11 (9–14)	11 (9–13)	0.868
CIRS-SI	1 (1–3)	1 (0–3)	1 (1–2)	0.835
Drugs, number	8 (5–10)	8 (5–10)	7 (5–9)	0.362
CFS	4 (3–6)	4 (3–6)	4 (4–6)	0.742
PC-FI	0.24 (0.16–0.36)	0.20 (0.12–0.36)	0.24 (0.16–0.40)	0.473
Blood tests upon admission				
Arterial pH	7.43 (7.40–7.47)	7.42 (7.34–7.43)	7.45 (7.42–7.47)	**0.002**
Bicarbonate, mmol/L	26 (23–31)	26 (23–30)	28 (23–31)	0.502
pCO_2_, mmHg	42 (35–48)	44 (39–47)	38 (33–50)	0.176
pO_2_, mmHg	67 (59–75)	67 (61–80)	67 (58–75)	0.684
P/F ratio	264 (196–328)	300 (219–329)	223 (180–323)	0.147
WBC, *n*/mm^3^	7995 (5940–10,640)	7630 (5460–10,230)	8520 (6160–11500)	0.284
Haemoglobin, g/dL	12.0 (10.7–13.9)	11.3 (10.6–13.6)	12.7 (11.2–13.9)	0.241
Creatinine, mg/dL	1.1 (0.8–1.6)	1.1 (0.9–1.5)	1.1 (0.8–1.9)	0.826
CRP, mg/L	37 (16–100)	40 (11–101)	37 (21–102)	0.634
Procalcitonin, ng/mL	0.16 (0.06–0.46)	0.19 (0.07–0.54)	0.13 (0.04–0.47)	0.613
Lung ultrasound characteristics				
LUS score on T0	11 (5–21)	5 (2–9)	20 (16–15)	**<0.001**
LUS score on T1	7 (4–9)	6 (3–8)	9 (6–15)	**0.002**
Delta LUS score T1–T0	−4 (−8–0)	0 (−3–2)	−8 (−15–−4)	**<0.001**
Diagnosis of pneumonia at T0, %	47	48	46	0.898
Diagnosis of other consolidation at T0, %	49	40	58	0.214
Diagnosis of effusion at T0, %	67	60	73	0.332
Diagnosis of interstitial syndrome at T0, %	90	80	100	**0.016**
Endpoints				
NIV or HFNC, %	12	12	11	1.000
Oxygen duration, days	5 (3–9)	6 (3–10)	5 (3–9)	0.813
Delirium, %	10	4	15	0.193
LOS, days	8 (6–13)	7 (6–11)	8 (6–14)	0.563
Hospital mortality, %	6	4	7	1.000
3-month readmissions, %	13	0	26	**0.010**

LUS = Lung ultrasound; T0 = Ultrasound evaluation performed within 24 h of admission; T1 = Ultrasound evaluation performed after 72 h; COPD = Chronic obstructive pulmonary disease; CKD = Chronic kidney disease; CIRS-CS = Cumulative Illness Rating Scale-Comorbidity Score; CIRS-SI = Cumulative Illness Rating Scale-Severity Index; CFS = Clinical Frailty Scale; PC-FI = Primary Care-Frailty Index; WBC = White blood cells; CRP = C-reactive protein; NIV = Non-invasive ventilation; HFNC = High-flow nasal cannula; LOS = Length of stay. Data expressed as median and IQR or percentage. *p* values calculated with Mann–Whitney test for continuous variables, Chi-square test or Fisher’s exact test for dichotomous variables. *p* < 0.05 indicated in bold.

**Table 2:** Diaphragm and right vastus lateralis muscle ultrasound findings in all study participants and comparison after stratification for LUS score median at T0 (within 24 h of admission).

Parameter	Overall Population (*N* = 52)	Patients with LUS Score < 11 at T0 (*N* = 25)	Patients with LUS Score ≥ 11 at T0 (*N* = 27)	*p*
Diaphragm ultrasound measurements				
Diaphragm excursion on quiet breathing, T0, mm	17.1 (12.2–22.6)	19.1 (15.7–25.0)	15.2 (11.4–21.1)	**0.023**
Diaphragm excursion on quiet breathing, T1, mm	17.8 (12.7–23.4)	20.5 (13.9–24.6)	17.6 (12.3–22.4)	0.367
Diaphragm excursion on MVI, T0, mm	34.7 (24.2–52.3)	45.4 (25.8–63.5)	31.7 (21.5–45.2)	0.056
Diaphragm excursion on MVI, T1, mm	41.5 (25.6–64.0)	41.8 (27.4–67.4)	39.6 (25.4–62.1)	0.903
Diaphragm thickness on TLC, T0, mm	8.5 (6.0–12.0)	8.5 (7.7–10.2)	7.7 (5.4–12.2)	0.833
Diaphragm thickness on TLC, T1, mm	9.3 (6.4–11.3)	9.5 (7.0–10.9)	8.4 (5.8–12.5)	0.697
Diaphragm thickness on TV, T0, mm	6.2 (4.3–8.0)	6.2 (4.5–8.0)	5.9 (3.8–8.8)	0.934
Diaphragm thickness on TV, T1, mm	5.9 (4.2–7.2)	6.0 (5.0–7.0)	5.7 (3.9–8.5)	0.879
Diaphragm thickness on FRC, T0, mm	4.4 (3.6–5.9)	4.3 (3.6–5.6)	4.5 (3.4–8.7)	0.741
Diaphragm thickness on FRC, T1, mm	4.4 (3.8–5.7)	4.3 (3.8–5.0)	4.4 (3.2–6.0)	0.606
Right vastus lateralis ultrasound measurements				
Vastus lateralis thickness, T0, mm	16.2 (12.5–20.7)	17.4 (14.5–20.0)	15.3 (11.3–20.8)	0.245
Vastus lateralis thickness, T1, mm	15.7 (12.3–18.5)	14.2 (11.8–17.7)	15.9 (12.8–19.3)	0.248
Vastus lateralis CSA, T0, cm^2^	9.2 (7.3–11.9)	9.1 (7.8–11.3)	9.2 (6.5–12.3)	0.615
Vastus lateralis CSA, T1, cm^2^	8.8 (7.0–11.5)	9.1 (7.5–11.7)	8.7 (6.2–10.6)	0.327

LUS = Lung ultrasound; MVI = Maximal voluntary inspiration; TLC = Total lung capacity; TV = Tidal volume; FRC = Functional residual capacity; CSA = Cross-sectional area. *p* < 0.05 are indicated in bold.

**Table 3:** Comparison of the main characteristics and outcomes of patients, stratified according to the median (17.1 mm) of diaphragmatic excursion on quiet breathing on T0 (within 24 h of hospitalization).

Parameter	Patients with Diaphragm Excursion on Quiet Breathing at T0 < 17.1 mm (*N* = 25)	Patients with Diaphragm Excursion on Quiet Breathing at T0 ≥ 17.1 mm (*N* = 25)	*p*
Age, years	84 (80–87)	84 (78–90)	0.865
Females, %	46	52	0.684
Weight, kg	74 (65–82)	68 (61–81)	0.503
Chronic illnesses, number	5 (4–6)	4 (4–5)	0.218
COPD, %	58	24	**0.014**
Heart disease, %	77	68	0.485
Obesity, %	35	24	0.416
CKD, %	27	28	0.933
Dementia, %	12	12	1.000
CIRS-CS	11 (9–12)	11 (8–14)	0.791
CIRS-SI	1 (1–2)	1 (0–3)	0.740
Drugs, number	9 (6–10)	8 (5–10)	0.256
CFS	4 (4–5)	4 (3–6)	0.486
PC-FI	0.28 (0.20–0.37)	0.16 (0.12–0.34)	**0.034**
Arterial pH	7.44 (7.42–7.48)	7.43 (7.34–7.45)	0.153
Bicarbonate, mmol/L	29 (24–31)	25 (23–28)	0.067
pCO_2_, mmHg	43 (36–51)	40 (34–47)	0.393
pO_2_, mmHg	65 (59–72)	70 (60–87)	0.195
P/F ratio	262 (190–316)	271 (199–329)	0.369
WBC, *n*/mm^3^	8525 (6460–11,238)	7780 (5265–10,385)	0.474
Haemoglobin, g/dL	13.2 (11.4–14.2)	11.2 (9.8–13.0)	**0.007**
Creatinine, mg/dL	1.0 (0.8–1.4)	1.1 (0.9–1.7)	0.411
CRP, mg/L	30 (18–91)	56 (14–169)	0.337
Procalcitonin, ng/mL	0.10 (0.04–0.37)	0.30 (0.08–0.98)	0.052
LUS score on T0	12 (6–21)	10 (4–19)	0.360
LUS score on T1	8 (6–10)	6 (2–10)	0.220
Delta LUS score T1–T0	−3 (−12–0)	−4 (−8–0)	0.482
Diagnosis of pneumonia, %	36	60	0.093
Diagnosis of other consolidation, %	52	48	0.783
Diagnosis of effusion, %	60	72	0.381
Diagnosis of interstitial syndrome, %	92	88	0.646
NIV or HFNC, %	12	12	0.960
Oxygen duration, days	5 (3–11)	5 (2–8)	0.206
Delirium during hospitalization, %	15	0	0.110
LOS, days	8 (5–14)	7 (6–12)	0.872
Hospital mortality, %	8	4	1.000
3-month readmissions, %	19	4	0.191

LUS = Lung ultrasound; T0 = Ultrasound evaluation performed within 24 h of admission; T1 = Ultrasound evaluation performed after 72 h; COPD = Chronic obstructive pulmonary disease; CKD = Chronic kidney disease; CIRS-CS = Cumulative Illness Rating Scale-Comorbidity Score; CIRS-SI = Cumulative Illness Rating Scale-Severity Index; CFS = Clinical Frailty Scale; PC-FI = Primary Care-Frailty Index; WBC = White blood cells; CRP = C-reactive protein; NIV = Non-invasive ventilation; HFNC = High-flow nasal cannula; LOS = Length of stay. Data expressed as median and IQR or percentage. *p* calculated with Mann–Whitney, Chi-square or Fisher’s exact tests. *p* < 0.05 are indicated in bold.

## Data Availability

The datasets presented in this article are not readily available because of ethical restrictions imposed by the Italian regulation on personal health data. Requests to access the datasets, in rigorously anonymous form, should be directed to the corresponding author.

## Supplementary Materials

The following supporting information can be downloaded at: https://www.mdpi.com/article/10.3390/diagnostics15010087/s1. Supplementary Table S1: Comparison of participants after stratification for 3-month readmissions. Supplementary Table S2: Comparison of the baseline characteristics and outcomes of study participants, stratified according to the median of LUS score at the ultrasound examination performed after 72 h of admission (T1). Supplementary Table S3: Comparison of the baseline characteristics and outcomes of study participants, stratified according variation of LUS score between the ultrasound examination performed within 24 h of admission (T0) and the one performed after 72 h (T1). Supplementary Table S4: Comparison of the baseline characteristics and outcomes of study participants, stratified according to the presence of novel diagnoses in the lung ultrasound examination performed on T1 (72 h from admission), in comparison with the examination performed on T0 (within 24 h of admission). Supplementary Table S5: Factors associated with different ultrasonographic measures of diaphragm excursion at different time points, determined with stepwise linear regression analysis. Supplementary Table S6: Results of receiver operating characteristics (ROC) analysis testing the capacity of ultrasound measures of diaphragm thickness upon admission (T0) and vastus lateralis muscle area after 72 h of hospitalization (T1) to discriminate between the condition of obesity the studied population. Supplementary Table S7: Comparison of the main characteristics and outcomes of participants categorized by vastus lateralis muscle cross-sectional area (CSA) cut-off for detecting obesity at T1 (72 h from hospital admission).
