# Supplementary material for: Integrated Lung, Diaphragm and Lower Limb Muscular Ultrasound: Clinical Correlations in Geriatric Patients with Acute Respiratory Illness
Source: Diagnostics (Basel). 2025 Jan 2;15(1):87. doi: 10.3390/diagnostics15010087 (PMC11719607; doi:10.3390/diagnostics15010087)
Supplement: Supplementary file 1 [file diagnostics-15-00087-s001.zip › diagnostics-3366027-supplementary.pdf]

SUPPLEMENTARY TABLE S1

Comparison of participants after stratification for 3-month readmissions.

Parameter	Patients with no 3-month readmissions (N=42)	Patients with 3-month readmissions (N=7)	p
Age, years	84 (81-89)	86 (74-87)	0.979
Females, %	49	43	1.000
Weight, kg	70 (64-81)	78 (56-90)	0.907
Chronic illnesses, number	4 (4-6)	5 (4-7)	0.461
COPD, %	38	57	0.420
Heart disease, %	71	86	0.659
Obesity, %	27	57	0.182
CKD, %	29	29	1.000
Dementia, %	11	14	1.000
CIRS-CS	11 (8-14)	12 (10-12)	0.636
CIRS-SI	1 (0-3)	1 (1-2)	0.636
Drugs, number	8 (5-10)	7 (7-9)	0.674
CFS	4 (3-6)	5 (4-5)	0.528
PC-FI	0.24 (0.14-0.36)	0.24 (0.20-0.40)	0.618
Arterial pH	7.43 (7.40-7.45)	7.47 (7.41-7.49)	0.145
Bicarbonate, mmol/l	26 (23-29)	31 (29-36)	**0.021**
pCO_2, mmHg	41 (34-46)	52 (36-58)	0.128
pO_2, mmHg	68 (59-79)	62 (58-69)	0.113
P/F ratio	270 (198-329)	207 (178-328)	0.400
WBC, n/mm^3	8010 (5960-11100)	7980 (5630-9950)	0.581
Haemoglobin, g/dl	11.6 (10.6-13.8)	13.0 (12.0-13.9)	0.167
Creatinine, mg/dl	1.1 (0.9-1.7)	0.9 (0.7-1.5)	0.384
CRP, mg/L	48 (19-112)	19 (11-35)	**0.039**
Procalcitonin, ng/ml	0.20 (0.08-0.52)	0.04 (0.03-0.10)	**0.004**
LUS score on T_0	10 (4-17)	25 (18-30)	**<0.001**
LUS score on T_1	7 (3-10)	6 (6-9)	0.891
Delta LUS score T_1-T_0	-3 (-6-0)	-17 (-24- -12)	**<0.001**
Diagnosis of pneumonia at T_0, %	52	14	0.103
Diagnosis of other consolidation at T_0, %	55	14	0.099
Diagnosis of effusion at T_0, %	68	57	0.673
Diagnosis of interstitial syndrome at T_0, %	89	100	1.000

LUS=Lung Ultrasound; T0=Ultrasound evaluation performed within 24 hours from admission; T1=Ultrasound evaluation performed after 72 hours; COPD=Chronic Obstructive Pulmonary Disease; CKD=Chronic Kidney Disease; CIRS-CS=Cumulative Illness Rating Scale-Comorbidity Score; CIRS-SI=Cumulative Illness Rating Scale-Severity Index; CFS=Clinical Frailty Scale; PC-FI=Primary Care-Frailty Index; WBC=White Blood Cells; CRP=C-reactive protein. Data expressed as median and IQR or percentage. P values calculated with Mann-Whitney test for continuous variables, chi-square test or Fisher's exact test for dichotomous variables. P<0.05 indicated in bold.

SUPPLEMENTARY TABLE S2

Comparison of the baseline characteristics and outcomes of study participants, stratified according to the median of LUS score at the ultrasound examination performed after 72 hours from admission (T_1).

Parameter	Patients with LUS score <7 at T_1 (N=22)	Patients with LUS score ≥7 at T_1 (N=28)	p
Age, years	84 (79-86)	86 (78-90)	0.150
Females, %	55	43	0.422
Weight, kg	74 (61-81)	70 (63-90)	0.909
Chronic illnesses, number	4 (4-6)	5 (4-6)	0.537
COPD, %	45	39	0.669
Heart disease, %	68	79	0.416
Obesity, %	36	25	0.394
CKD, %	14	43	**0.025**
Dementia, %	18	7	0.385
CIRS-CS	10 (8-13)	12 (10-14)	0.077
CIRS-SI	1 (0-2)	2 (1-3)	0.155
Drugs, number	8 (5-10)	8 (5-10)	0.961
CFS	4 (3-5)	5 (3-6)	0.522
PC-FI	0.20 (0.12-0.28)	0.30 (0.20-0.40)	**0.013**
Arterial pH	7.43 (7-40-7.45)	7.44 (7.41-7.47)	0.660
Bicarbonate, mmol/l	27 (25-30)	26 (23-31)	0.462
pCO_2, mmHg	43 (36-52)	43 (34-48)	0.545
pO_2, mmHg	66 (62-74)	69 (59-80)	0.627
P/F ratio	271 (193-329)	237 (191-326)	0.515
WBC, n/mm^3	7635 (5585-10175)	8940 (6490-11413)	0.328
Haemoglobin, g/dl	11.9 (10.5-13.8)	12.5 (10.8-13.9)	0.625
Creatinine, mg/dl	1.0 (0.7-1.3)	1.2 (0.9-2.0)	0.071
CRP, mg/L	26 (11-81)	63 (26-162)	**0.039**
Procalcitonin, ng/ml	0.08 (0.04-0.28)	0.32 (0.12-0.81)	**0.043**
LUS score on T_0	7 (3-12)	16 (10-22)	**0.007**
LUS score on T_1	3 (1-6)	9 (8-16)	**<0.001**
Delta LUS score T_1-T_0	-4 (-8-0)	-3 (-9-0)	0.776
Diagnosis of pneumonia, %	41	56	0.318
Diagnosis of other consolidation, %	36	59	0.115
Diagnosis of effusion, %	68	63	0.710
Diagnosis of interstitial syndrome, %	82	96	0.100
NIV or HFNC, %	5	18	0.211
Oxygen duration, days	5 (2-9)	6 (3-10)	0.281
LOS, days	8 (6-10)	9 (6-14)	0.276
Hospital mortality, %	0	11	0.246
3-month readmissions, %	18	11	0.684

LUS=Lung Ultrasound; T_0=Ultrasound evaluation performed within 24 hours from admission; T_1=Ultrasound evaluation performed after 72 hours; COPD=Chronic Obstructive Pulmonary Disease; CKD=Chronic Kidney Disease; CIRS-CS=Cumulative Illness Rating Scale-Comorbidity Score; CIRS-SI=Cumulative Illness Rating Scale-Severity Index; CFS=Clinical Frailty Scale; PC-FI=Primary Care-Frailty Index; WBC=White Blood Cells; CRP=C-reactive protein; NIV=Non-Invasive Ventilation; HFNC= High-Flow Nasal Cannula; LOS=Length of Stay.Data expressed as median and IQR or percentage. P values calculated with Mann-Whitney for

continuous variables, for dichotomous variables chi-square test or Fisher's exact test, the latter in the presence of cells with expected value less than 5. $P<0.05$ are indicated in bold.

SUPPLEMENTARY TABLE S3

Comparison of the baseline characteristics and outcomes of study participants, stratified according variation of LUS score between the ultrasound examination performed within 24 hours from admission (T_0) and the one performed after 72 hours (T_1).

Parameter	Patients with LUS score T_1-T_0 <0 (improvement) (N=33)	Patients with LUS score T_1-T_0 ≥0 (unchanged or worsening) (N=17)	p
Age, years	84 (79-88)	84 (78-91)	0.719
Females, %	48	47	0.926
Weight, kg	69 (61-81)	71 (63-87)	0.771
Chronic illnesses, number	5 (4-6)	4 (3-6)	0.144
COPD, %	45	35	0.500
Heart disease, %	76	71	0.700
Obesity, %	33	24	0.484
CKD, %	30	29	0.949
Dementia, %	3	29	**0.014**
CIRS-CS	11 (8-13)	11 (9-14)	0.853
CIRS-SI	1 (1-2)	1 (0-3)	0.680
Drugs, number	8 (5-9)	9 (5-11)	0.360
CFS	4 (3-5)	6 (3-7)	0.240
PC-FI	0.24 (0.16-0.30)	0.32 (0.16-0.40)	0.149
WBC, n/mm^3	7980 (6205-12240)	9500 (4855-12820)	0.645
Haemoglobin, g/dl	12.0 (10.8-14.0)	12.0 (10.3-13.8)	0.720
Creatinine, mg/dl	1.1 (0.9-1.7)	1.1 (0.8-1.6)	0.704
CRP, mg/L	38 (19-104)	47 (14-136)	0.931
Procalcitonin, ng/ml	0.14 (0.04-0.47)	0.19 (0.08-0.47)	0.516
LUS score on T_0	16 (10-23)	5 (1-9)	**<0.001**
LUS score on T_1	7 (4-10)	8 (4-13)	0.688
Delta LUS score T_1-T_0	-6 (-13- -4)	0 (0-4)	**<0.001**
Diagnosis of pneumonia, %	50	47	0.849
Diagnosis of other consolidation, %	44	59	0.325
Diagnosis of effusion, %	69	59	0.497
Diagnosis of interstitial syndrome, %	100	71	**<0.001**
Diaphragm excursion on quiet breathing, T_0, mm	16.2 (11.7-22.2)	20.0 (15.3-25.0)	0.162
Diaphragm excursion on quiet breathing, T_1, mm	17.1 (12.7-21.9)	21.4 (14.1-25.8)	0.260
Diaphragm excursion on MVI, T_0, mm	32.4 (23.6-50.9)	36.9 (24.6-52.7)	0.653
Diaphragm excursion on MVI, T_1, mm	41.8 (25.8-63.8)	41.2 (18.4-71.0)	0.860
NIV or HFNC, %	9	18	0.388
Oxygen duration, days	5 (2-9)	6 (4-11)	0.282
LOS, days	8 (6-14)	7 (6-12)	0.886
Hospital mortality, %	6	6	1.000
3-month readmissions, %	21	0	0.080

LUS=Lung Ultrasound; T_0=Ultrasound evaluation performed within 24 hours from admission; T_1=Ultrasound evaluation performed after 72 hours; COPD=Chronic Obstructive Pulmonary Disease; CKD=Chronic Kidney Disease; CIRS-CS=Cumulative Illness Rating Scale-Comorbidity Score; CIRS-SI=Cumulative Illness Rating Scale-Severity Index; CFS=Clinical Frailty Scale; PC-FI=Primary Care-Frailty Index; WBC=White Blood Cells;

CRP=C-reactive protein; MVI=Maximal Voluntary Inspiration; NIV=Non-Invasive Ventilation; HFNC = High-Flow Nasal Cannula; LOS=Length of Stay.

Data expressed as median and IQR or percentage. P values calculated with Mann-Whitney for continuous variables, for dichotomous variables chi-square test or Fisher's exact test, the latter in the presence of cells with expected value less than 5. P<0.05 are indicated in bold.

SUPPLEMENTARY TABLE S4

Comparison of the baseline characteristics and outcomes of study participants, stratified according to the presence of novel diagnoses in the lung ultrasound examination performed on T_1 (72 hours from admission), in comparison with the examination performed on T_0 (within 24 hours from admission).

Parameter	Patients without novel diagnoses on T_1 (N=38)	Patients with novel diagnoses on T_1 (N=12)	p
Age, years	86 (77-89)	84 (82-88)	0.673
Females, %	55	33	0.193
Weight, kg	74 (62-88)	68 (61-78)	0.485
Chronic illnesses, number	5 (4-6)	4 (3-7)	0.595
COPD, %	47	25	0.171
Heart disease, %	71	83	0.480
Obesity, %	34	25	0.728
CKD, %	29	33	1.000
Dementia, %	11	17	0.621
CIRS-CS	11 (8-13)	13 (9-15)	0.338
CIRS-SI	1 (0-2)	3 (1-4)	**0.036**
Drugs, number	8 (5-9)	10 (7-11)	0.205
CFS	4 (3-6)	5 (4-6)	0.328
PC-FI	0.24 (0.16-0.31)	0.26 (0.20-0.39)	0.681
WBC, n/mm^3	8265 (5823-10633)	7905 (6350-10525)	0.865
Haemoglobin, g/dl	12.4 (10.7-13.9)	11.4 (11.1-13.2)	0.562
Creatinine, mg/dl	1.1 (0.9-1.7)	1.1 (0.7-1.5)	0.419
CRP, mg/L	38 (14-96)	48 (21-242)	0.477
Procalcitonin, ng/ml	0.14 (0.06-0.45)	0.20 (0.04-0.08)	0.852
LUS score on T_0	11 (6-21)	12 (2-20)	0.532
LUS score on T_1	7 (5-10)	6 (3-9)	0.717
Delta LUS score T_1-T_0	-4 (-8-0)	-4 (-12-2)	0.806
NIV or HFNC, %	13	8	1.000
Oxygen duration, days	5 (2-8)	8 (4-12)	0.086
LOS, days	7 (5-11)	10 (7-13)	**0.047**
Hospital mortality, %	5	0	1.000
3-month readmissions, %	13	17	1.000

LUS=Lung Ultrasound; T0=Ultrasound evaluation performed within 24 hours from admission; T1=Ultrasound evaluation performed after 72 hours; COPD=Chronic Obstructive Pulmonary Disease; CKD=Chronic Kidney Disease; CIRS-CS=Cumulative Illness Rating Scale-Comorbidity Score; CIRS-SI=Cumulative Illness Rating Scale-Severity Index; CFS=Clinical Frailty Scale; PC-FI=Primary Care-Frailty Index; WBC=White Blood Cells; CRP=C-reactive protein; NIV=Non-Invasive Ventilation; HFNC = High-Flow Nasal Cannula; LOS=Length of Stay. Data expressed as median and IQR or percentage. P values calculated with Mann-Whitney for continuous variables, for dichotomous variables chi-square test or Fisher's exact test, the latter in the presence of cells with expected value less than 5. P<0.05 are indicated in bold.

SUPPLEMENTARY TABLE S5

Factors associated with different ultrasonographic measures of diaphragm excursion at different time points, determined with stepwise linear regression analysis.

**PARAMETER**	**BETA ± SE**	**Standardized BETA**	**P**
Model 1: Factors associated with diaphragm excursion on quiet breathing at T_0			
Haemoglobin, g/dl	-1.643 ± 0.432	-0.455	**<0.001**
COPD or heart failure	-5.547 ± 2.036	-0.325	**0.009**
Model 2: Factors associated with diaphragm excursion on maximal voluntary inspiration at T_0			
Diaphragm excursion on quiet breathing at T_0	1.862 ± 0.442	0.556	**<0.001**
Procalcitonin, ng/ml	1.977 ± 0.769	0.344	**0.014**
Model 3: Factors associated with diaphragm excursion on quiet breathing at T_1			
Diaphragm excursion on maximal voluntary inspiration at T_0	0.261 ± 0.046	0.660	**<0.001**
Model 4: Factors associated with diaphragm excursion on maximal voluntary inspiration at T_1			
Diaphragm excursion on quiet breathing at T_1	1.295 ± 0.331	0.475	**<0.001**
Procalcitonin, ng/ml	2.375 ± 0.646	0.447	**0.001**
PC-FI	-55.949 ± 23.122	-0.285	**0.023**

T_0=Ultrasound evaluation performed within 24 hours from admission; T_1=Ultrasound evaluation performed after 72 hours; COPD=Chronic Obstructive Pulmonary Disease; PC-FI=Primary Care-Frailty Index.

SUPPLEMENTARY TABLE S6

Results of Receiver Operating Characteristics (ROC) analysis testing the capacity of ultrasound measures of diaphragm thickness upon admission (T_0) and vastus lateralis muscle area after 72 hours of hospitalization (T_1) to discriminate between the condition of obesity the studied population.

**ULTRASOUND PARAMETER**	**AUC**	**95% CI**	**P**	**CUT-OFF (Youden test)**	**Sensitivity**	**Specificity**
Diaphragm thickness on TV, T_0, mm	0.819	0.652-0.987	**<0.001**	7.25 mm	87.5	66.7
Diaphragm thickness on FRC, T_0, mm	0.792	0.589-0.994	**0.005**	5.52 mm	75.0	83.3
Diaphragm thickness on TLC, T_0, mm	0.753	0.529-0.978	**0.027**	16.26 mm	52.5	94.4
Vastus lateralis CSA, T_1, cm^2	0.910	0.798-0.982	**<0.001**	9.91 cm^2	87.5	83.3

AUC=Area Under the ROC Curve; CI=Confidence Interval; TV=Tidal Volume; FRC=Functional Residual Capacity; TLC=Total Lung Capacity; CSA=Cross-Sectional Area. P values <0.05 are indicated in bold.

SUPPLEMENTARY TABLE S7

Comparison of the main characteristics and outcomes of participants categorized by vastus lateralis muscle cross-sectional area (CSA) cut-off for detecting obesity at T_1 (72 h from hospital admission).

Parameter	Patients with right vastus lateralis CSA at T_1 <9.91 cm^2 (N=30)	Patients with right vastus lateralis CSA at T_1 ≥9.91 cm^2 (N=20)	p
Age, years	85 (83-89)	82 (75-87)	0.175
Females, %	57	37	0.183
Weight, kg	66 (57-61)	81 (77-92)	**<0.001**
COPD, %	47	37	0.508
Heart disease, %	80	63	0.201
Obesity, %	10	58	**<0.001**
CKD, %	27	32	0.718
Dementia, %	17	5	0.384
CIRS-CS	12 (9-14)	10 (8-11)	**0.020**
CIRS-SI	2 (1-3)	1 (0-2)	0.077
CFS	5 (4-6)	3 (3-4)	<**0.001**
PC-FI	0.28 (0.19-0.40)	0.20 (0.12-0.24)	**0.010**
P/F upon admission, mmHg	223 (184-306)	324 (226-344)	**0.031**
Diaphragm excursion on quiet breathing, T_0, mm	16.6 (12.2-22.0)	20.0 (11.4-25.9)	0.207
Diaphragm excursion on quiet breathing, T_1, mm	17.6 (12.3-21.5)	17.9 (13.2-31.8)	0.157
Diaphragm excursion on maximal voluntary inspiration, T_0, mm	27.7 (22.7-47.0)	40.2 (32.2-70.0)	0.066
Diaphragm excursion on maximal voluntary inspiration, T_1, mm	39.6 (22.1-50.9)	59.1 (32.9-76.2)	**0.037**
Diaphragm thickness on TLC, T_0, mm	7.7 (5.3-9.9)	9.7 (8.1-22.1)	**0.024**
Diaphragm thickness on TLC, T_1, mm	7.9 (5.7-9.8)	9.8 (8.9-14.6)	**0.005**
Diaphragm thickness on TV, T_0, mm	5.5 (3.6-7.3)	7.6 (5.5-14.8)	**0.008**
Diaphragm thickness on TV, T_1, mm	5.3 (3.9-6.8)	7.0 (5.9-9.5)	**0.004**
Diaphragm thickness on FRC, T_0, mm	4.4 (3.6-5.5)	5.5 (4.0-12.7)	0.080
Diaphragm thickness on FRC, T_1, mm	3.9 (3.2-4.8)	5.0 (4.3-9.1)	**0.013**
Oxygen duration, days	7 (4-11)	4 (0-7)	**0.008**
LOS, days	9 (7-13)	7 (5-10)	0.125
Hospital mortality, %	10	0	0.273
3-month readmissions, %	13	11	1.000

COPD=Chronic Obstructive Pulmonary Disease; CKD=Chronic Kidney Disease; CIRS-CS=Cumulative Illness Rating Scale-Comorbidity Score; CIRS-SI=Cumulative Illness Rating Scale-Severity Index; CFS=Clinical Frailty Scale; PC-FI=Primary Care-Frailty Index; TLC=Total Lung Capacity; TV=Tidal Volume; FRC=Functional Residual Capacity; LOS=Length of Stay.

Data expressed as median and IQR or percentage. P values calculated with Mann-Whitney test for continuous variables, chi-square test or Fisher's exact test for dichotomous variables. P<0.05 indicated in bold.
